# Ligation of TLR Homologue CD180 of B Cells Activates the PI3K/Akt/mTOR Pathway in Systemic Sclerosis and Induces a Pathological Shift in the Expression of BAFF Receptors

**DOI:** 10.3390/ijms23126777

**Published:** 2022-06-17

**Authors:** Szabina Erdő-Bonyár, Judit Rapp, Dávid Szinger, Tünde Minier, Gábor Kumánovics, László Czirják, Timea Berki, Diána Simon

**Affiliations:** 1 Department of Immunology and Biotechnology, Clinical Center, University of Pécs Medical School, 7624 Pécs, Hungary; erdo-bonyar.szabina@pte.hu (S.E.-B.); rapp.judit@pte.hu (J.R.); szinger.david96@gmail.com (D.S.); simon.diana@pte.hu (D.S.); 2 Department of Rheumatology and Immunology, Clinical Center, University of Pécs Medical School, 7632 Pécs, Hungary; minier.tunde@pte.hu (T.M.); kumanovics.gabor@pte.hu (G.K.); czirjak.laszlo@pte.hu (L.C.)

**Keywords:** CD180, TLR, B cells, PI3K/Akt, mTOR, BAFF-R, TACI, anti-BAFF autoantibody, systemic sclerosis

## Abstract

The phosphatidylinositol-3-kinase (PI3K)/Akt and the mammalian target of rapamycin (mTOR) pathways are known to play a key role in B-cell activation and fibrosis in systemic sclerosis (SSc). Receptors of B-cell activator factor (BAFF) utilize these pathways, which can be influenced by Toll-like receptors (TLRs), as TLRs can alter the expression of BAFF-binding receptors. Our results show that B-cell stimulation via TLR homologue CD180 phosphorylates Akt in diffuse cutaneous SSc (dcSSc) to a lower extent than in healthy controls (HCs). We found basal downregulated BAFF receptor (BAFF-R) and enhanced transmembrane activator and calcium-modulator and cyclophilin ligand interactor (TACI) expression in dcSSc B cells, which might enhance the formation of autoantibody-secreting plasma cells. Moreover, this pathological shift was observed in naive B cells, emphasizing the importance of their increase in SSc. Additionally, we measured higher serum levels of autoantibodies to BAFF in dcSSc patients, suggesting that an imbalance in the complex system of BAFF/anti-BAFF autoantibodies/BAFF-binding receptors may contribute to the development of SSc. Anti-CD180 antibody treatment had opposite effects on the expression of BAFF-R and TACI in HC B cells, resulting in similar levels as observed in SSc B cells without stimulation, which argues against the usefulness of such therapy in SSc.

## 1. Introduction

The phosphatidylinositol-3-kinase (PI3K)/Akt and the mammalian target of rapamycin (mTOR) signaling pathways have been proposed to be implicated to play a crucial role in the pathogenesis of systemic sclerosis (SSc)—in particular, in two critical aspects, fibrogenesis and B-cell activation [1,2], which are two typical features of SSc [3]. The PI3K/Akt/mTOR signaling pathway has a central role in many aspects of cell growth and survival [4]. Hyperactivity of PI3K/Akt/mTOR/S6 signaling has been found in the dermal fibroblasts of SSc patients [1] and inhibition of this pathway leads to significant protection against TGFβ-induced fibroblast growth in an SSc mouse model [1,5,6]. The central role of the PI3K/Akt/mTOR axis has also been shown in B-cell activation [7], and B-cell activator factor (BAFF) was also linked to the PI3K/Akt/mTOR pathway in lupus nephritis [8]. 

BAFF is an important factor for B-cell proliferation and maturation and the increased amount of BAFF may contribute to the development of autoimmune disorders [8,9]. Overexpression of BAFF produced a lupus- and Sjögren-like syndrome in mice [9] and elevated levels of BAFF have been described in several autoimmune disorders, including SSc [10]. Autoantibodies against BAFF have been described in healthy controls (HCs), but elevated levels in systemic lupus erythematosus (SLE) patients may contribute to autoimmune processes by regulating the availability and activity of BAFF [11,12]. BAFF can bind to multiple receptors on B cells, such as the BAFF receptor (BAFF-R) and transmembrane activator and calcium modulator and cyclophilin ligand interactor (TACI). BAFF-R is important in the survival and differentiation of B cells [9,13], while TACI has an essential role in isotype switching and plasma cell differentiation [9,14]. 

Toll-like receptors (TLRs) can influence the expression and signaling of BAFF receptors [15,16]. Binding of BAFF activates NF-κB signaling [14], and we previously showed that ligation of the TLR homologue CD180 induces the phosphorylation of NF-κB in B cells to a different extent in diffuse cutaneous SSc (dcSSc) and HCs [17]. Stimulation via CD180 leads to the activation of the vast majority of B cells [18]. Previously, we reported decreased expression of CD180 in the B cells of dcSSc patients [19], and CD180 was described to utilize the PI3K/Akt pathway in B-cell chronic lymphocytic leukemia (B-CLL) [20]. Consequently, the purpose of this study was to investigate the effects of CD180 ligation on the PI3K/Akt/mTOR pathway and the expression of BAFF receptors in peripheral blood B cells in dcSSc patients compared to HCs. An additional goal was to measure the serum levels of anti-BAFF antibodies to assess whether the complex system of anti-BAFF antibodies and BAFF-binding receptors regulating BAFF signaling could contribute to the development of SSc.

## 2. Results

### 2.1. Stimulation via CD180 Induces the Phosphorylation of Akt to a Lower Extent in dcSSc B Cells

**Figure 1:**
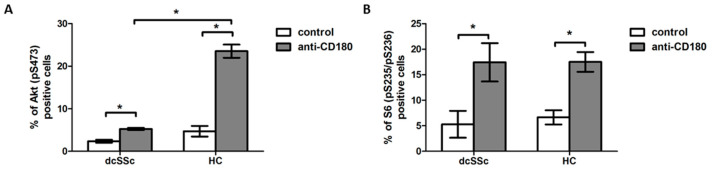
Induction of phosphorylation of Akt and S6 by CD180 ligation. Changes in the phosphorylation of Akt (pS473) (**A**) and S6 (pS235/pS236) (**B**) molecules in purified B cells of diffuse cutaneous SSc (dcSSc) patients (*n* = 6) and healthy controls (HCs) (*n* = 7) after stimulation with anti-CD180 antibody or left unstimulated (control) for 30 min, detected by flow cytometry. Data are presented as means ± SEM. * *p* < 0.05.

First, we investigated the effect of stimulation via CD180 on the activation of Akt, a dominant effector molecule of PI3K [21], in separated B cells of dcSSc patients and HCs, and found that the CD180 ligation significantly increased the percentage of phosphorylated Akt (pS473)-positive B cells in both dcSSc and HCs compared to unstimulated controls. However, the phosphorylation of Akt in the anti-CD180 antibody stimulated B cells was significantly lower in dcSSc than in HCs (Figure 1A). Since PI3K/Akt is a major activator of mTOR and S6 is a substrate of mTOR [22], we also measured the changes in the phosphorylation of S6. Stimulation via CD180 enhanced the ratio of phosphorylated S6 (pS235/pS236)-positive B cells to a similar extent in dcSSc and HCs (Figure 1B). 

### 2.2. Anti-BAFF Autoantibody Serum Level Is Higher in dcSSc 

**Figure 2:**
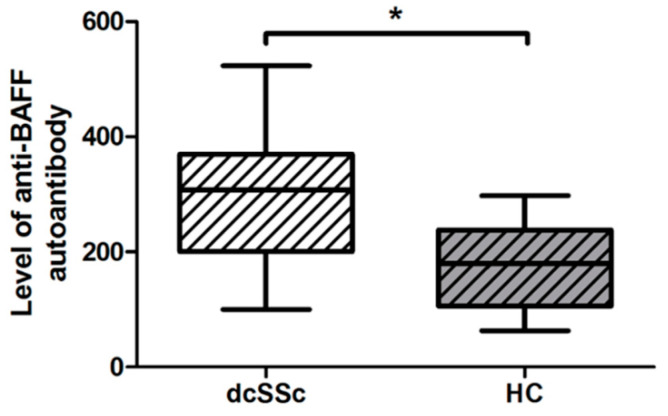
Difference in the serum level of autoantibody directed against BAFF in dcSSc (*n* = 20) and HCs (*n* = 21), measured by Luminex MAGPIX. The boxes show interquartile ranges (IQR), the horizontal lines represent medians, and the whiskers indicate the lowest and highest values. * *p* < 0.05.

BAFF utilizes the PI3K/Akt/mTOR/S6 signaling pathway [9] and BAFF is proposed to play a crucial role in the B-cell dysfunction in SSc [10]. The effect of elevated BAFF is reported to be regulated by the presence of autoantibodies directed against BAFF [11]; therefore, we compared the levels (mean fluorescence intensity, MFI) of autoantibodies against BAFF in the serum samples of dcSSc patients and healthy subjects. We found that the anti-BAFF autoantibody level was significantly higher in dcSSc than in HCs (Figure 2). 

### 2.3. Decreased Basal Expression of BAFF-R Is Accompanied by Elevated Expression of TACI in dcSSc B Cells

**Figure 3:**
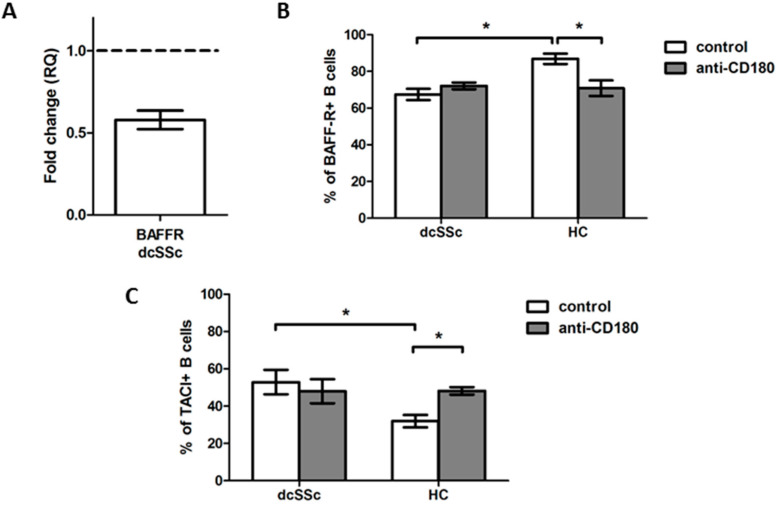
Analysis of BAFF-R and TACI expression in dcSSc and HC total B cells. (**A**) BAFF-R mRNA expression in total B cells of dcSSc patients (*n* = 3) compared to HCs (*n* = 3). Gene expression was normalized to HCs and the horizontal line (value 1) represents the expression of control samples. Changes in gene expression are shown as relative quantification (RQ) values. Data are shown as mean ± standard error of the mean (SEM). Alterations in the protein expression of BAFF-R (**B**) and TACI (**C**) in anti-CD180 antibody stimulated and unstimulated dcSSc (*n* = 4) and HC (*n* = 4) total B cells, as measured by flow cytometry. Data are presented as means ± SEM. * *p* < 0.05.

As BAFF exerts its effects via different receptors [9], first, we analyzed the expression of BAFF-R in purified B cells of dcSSc and HCs. The BAFF-R mRNA expression was downregulated in dcSSc compared to HC B cells (Figure 3A). Next, we examined the protein expression of BAFF-R in B cells in PBMC samples of dcSSc patients and HCs with flow cytometry. The results were similar to what we found when analyzing the BAFF-R mRNA expression; the protein level of BAFF-R was significantly lower in dcSSc than in HC B cells (Figure 3B). 

TLRs are reported to alter the expression of the receptors of BAFF [15,16]; thus, we also investigated whether stimulation of B cells via CD180 has different effects on the protein expression of BAFF-R in dcSSc and HCs. The ligation of CD180 significantly decreased the percentage of BAFF-R-positive B cells only in HCs, to the levels of unstimulated and stimulated dcSSc B cells. We found that the expression of BAFF-R was lower in total B cells in dcSSc than in HCs, but the stimulation via CD180 had no effect on the BAFF-R expression of dcSSc B cells (Figure 3B); therefore, we examined the expression of TACI in the total B cells of dcSSc and HCs. The percentage of TACI-positive B cells was higher in the unstimulated dcSSc than in HCs. Moreover, the anti-CD180 antibody stimulation significantly increased the TACI expression of B cells only in HCs, reaching the levels of unstimulated and stimulated dcSSc B cells (Figure 3C).

### 2.4. Basal Expression of BAFF-R Is Higher in HC Naive B Cells and Is Reduced by Anti-CD180 Antibody Treatment to the Level of dcSSc Naive B Cells 

**Figure 4:**
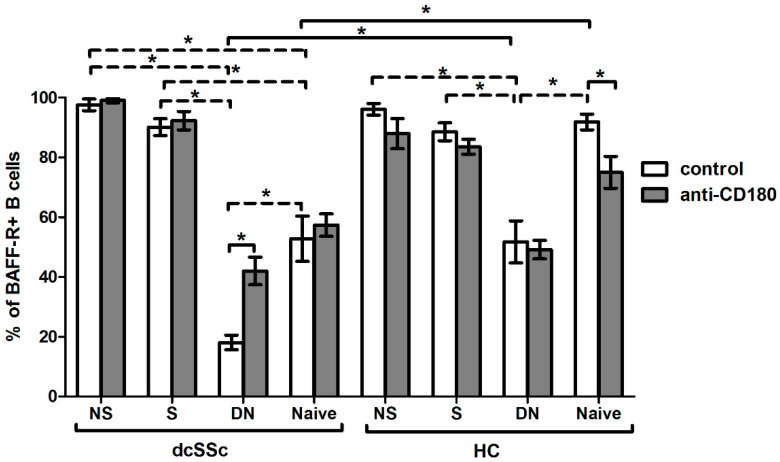
Effect of CD180 stimulation on BAFF-R expression of B cell subpopulations in dcSSc (*n* = 4) and HCs (*n* = 4). The percentage of BAFF-R-positive B cells in the four investigated B cell subsets, defined by CD27 and IgD labeling: CD27+IgD+ non-switched memory (NS), CD27+IgD- switched memory (S), CD27-IgD- double negative (DN) and CD27-IgD+ naive B cell subsets after anti-CD180 antibody stimulation or left unstimulated (control). The solid lines show significant differences between dcSSc patients and HCs, and between unstimulated and stimulated conditions, while the dashed lines indicate significant differences between unstimulated B cell subsets within groups. Data are presented as means ± SEM. * *p* < 0.05.

As we found that the expression of BAFF-R was lower in total B cells in dcSSc than in HCs, we investigated the expression of BAFF-R in B cell subsets. We analyzed the following B cell subpopulations defined by CD27 and IgD staining: CD27+IgD+ non-switched memory (NS), CD27+IgD- switched memory (S), CD27-IgD- double negative (DN) and CD27-IgD+ naive B cells. We compared the expression of BAFF-R of B cell subgroups between dcSSc and HCs and found that the basal BAFF-R expression was significantly lower in naive and DN B cells in dcSSc than HCs. Then, we analyzed the differences in the percentage of BAFF-R-positive cells in the unstimulated B cell subsets between the studied groups. We found that the ratio of BAFF-R-positive cells was the lowest in DN B cells compared to all other subsets, namely NS, S and naive B cells both in dcSSc and HCs. The expression of BAFF-R was significantly decreased in DN and naive B cells compared to memory B cells in dcSSc and was lower in DN B cells compared to memory and naive B cells in HCs. Next, we examined the effect of stimulation with anti-CD180 antibody on the expression of BAFF-R. Ligation via CD180 significantly increased the BAFF-R-positive cells in the DN B cells of dcSSc patients and significantly decreased it in the naive B cells of HCs. However, between dcSSc and HC samples, no differences were observed in the BAFF-R expression of any investigated B cell subsets after anti-CD180 antibody stimulation (Figure 4).

### 2.5. Basal Expression of TACI Is Lower in HC Naive B Cells, and Is Increased with Ligation of CD180 to the Level of dcSSc Naive B Cells 

**Figure 5:**
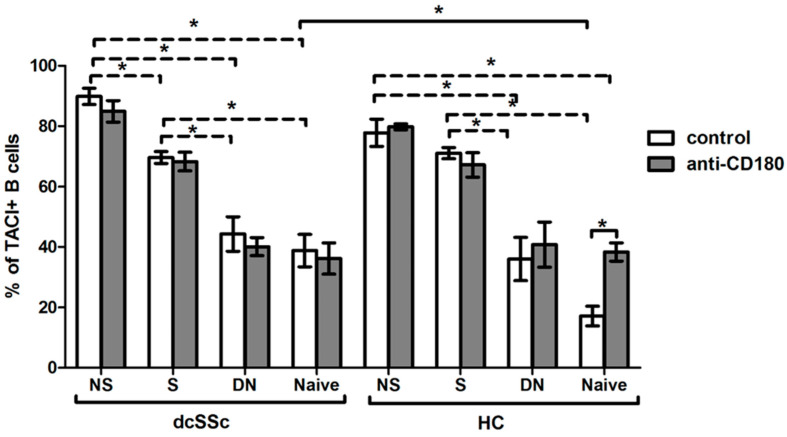
Changes in the TACI expression of B cell subsets in dcSSc (*n* = 4) and HCs (*n* = 4) after CD180 stimulation. The ratio of TACI-positive B cells in CD27+IgD+ non-switched memory (NS), CD27+IgD- switched memory (S), CD27-IgD- double negative (DN) and CD27-IgD+ naive B cell subsets with anti-CD180 antibody stimulation or left unstimulated. The solid lines show significant differences between dcSSc patients and HCs, and between unstimulated and stimulated conditions, while the dashed lines indicate significant differences between unstimulated B cell subsets within groups. Data are presented as means ± SEM. * *p* < 0.05.

We found a higher percentage of TACI-positive total B cells in dcSSc than in HCs; thus, we investigated the expression of TACI in the four described B cell subsets, namely NS, S, DN and naive, and found that the ratios of TACI-positive cells in the B cell subsets were similar between dcSSc and HCs, except in the naive cells, where the frequency of TACI-positive cells was significantly higher in dcSSc than in HCs under the unstimulated condition. Then, we examined the differences between the unstimulated B cell subsets within the investigated groups, and our results showed that the expression of TACI was higher in memory B cells, especially in NS B cells, compared to DN and naive cells both in dcSSc and HCs. Stimulation with anti-CD180 antibody did not change the expression of TACI in the B cell subsets of any groups, except in HC naive cells, where ligation of CD180 increased the level of TACI (Figure 5). 

## 3. Discussion

The activation of PI3K/Akt/mTOR signaling is involved in the fibrogenesis in SSc and inhibition of this axis has been shown to exert anti-fibrotic effects in an SSc mouse model [1,5,23]. According to a single-blind pilot study, the modified Rodnan skin score (MRSS) of SSc patients decreased after rapamycin treatment [24]. The involvement of the PI3K/Akt/mTOR pathway has also been shown in B-cell activation and differentiation [2], and it is evident that B cells play an essential role in the pathogenesis of SSc [3]. Moreover, TLR signaling can also act through this pathway [25], and elevated Akt phosphorylation has been described in HC and CD180-Responder B-CLL B cells after CD180 ligation [26]. Here, we demonstrated that stimulation via CD180 induced the phosphorylation of Akt and S6 both in dcSSc and HC B cells, but the activation of Akt was weaker in dcSSc than HCs, suggesting an impairment of the CD180 signaling regarding the PI3K/Akt pathway in dcSSc B cells. Forestier et al. [27] described a decrease in mTOR phosphorylation in the B cells of SSc patients compared to HCs. Our results did not show any differences in S6 phosphorylation between dcSSc and HC B cells, suggesting that alterations in the mTOR pathway in SSc B cells may influence other downstream molecules besides S6. 

The PI3K/Akt/mTOR pathway also plays an important role in BAFF-mediated B-cell differentiation and activation [8,28]. BAFF is essential to B-cell survival, maturation and homeostasis, and excessive amounts of BAFF could potentially contribute to the breakdown of B-cell tolerance by rescuing low-affinity self-reactive B cells from deletion, which may play a key role in the development of systemic autoimmune diseases [9,29]. Elevated BAFF serum levels were found in SSc and were associated with the severity and activity of the disease [10]. Although autoantibodies against BAFF were detected in HCs, the serum levels of anti-BAFF autoantibodies were higher in SLE patients than HCs [12,30]. Investigating the amount of anti-BAFF autoantibodies in SSc patients, we found similar results as dcSSc patients had higher levels of anti-BAFF autoantibodies than HCs. Consequently, the increased levels of autoantibodies against BAFF may be a regulatory mechanism in response to the elevated BAFF levels observed in SSc.

BAFF can bind to different receptors expressed on B cells, including BAFF-R and TACI [9]. BAFF-R is a positive regulator of B-cell homeostasis as it provides constitutive signals essential for B-cell development and survival [31]. TACI can enhance immunoglobulin class switching and the differentiation and survival of plasma cells [15]. Decreased BAFF-R expression and increased, but also unaltered, TACI expression was observed in SLE patients compared to HCs [32,33]. In this study, we found lower BAFF-R expression at mRNA and protein level and higher TACI expression at protein level in total B cells in dcSSc compared to HCs. The downregulation of BAFF-R and the enhanced TACI expression in SSc B cells may contribute to the differentiation of autoreactive B cells into autoantibody-secreting plasma cells [15,34], and autoantibodies are well known to play a crucial role in the pathogenesis of SSc [35]. 

BAFF-R is expressed by almost all B cell subsets, while TACI is expressed mainly by memory B cells and plasma cells, and mostly by activated cells, since TACI is an inducible receptor [14]. Darce et al. [34] found that human naive B cells have low TACI expression, but activation via TLR9 induces a rapid increase in TACI expression. According to our results, the differences in BAFF-R and TACI expression of total B cells between dcSSc patients and HCs could be due to the changes in the expression of naive B cells, since we found decreased expression of BAFF-R and augmented expression of TACI in naive B cells. The relevance of these findings is underlined by the elevated amount of naive B cells observed in SSc [36,37], and these results provide further evidence that differences in the distribution of B cell subsets—in particular, the increase in naive B cells—may play an important role in the development of SSc.

Altered expression of TLRs has been found to be associated with vascular damage in autoimmune diseases [38], and TLR signaling can influence the expression of BAFF receptors; TLR4, TLR7 and TLR9 stimulation resulted in the upregulated expression of TACI [15,16], but the changes observed in the expression of BAFF-R after B-cell activation via TLR4 and TLR9 vary between studies [38,39]. Activation via TLR9 was suggested to make B cells more sensitive to the effects of BAFF both in humans and mice, through the upregulation of TACI [16,38]. We showed that stimulation via CD180 resulted in a decrease in BAFF-R and an increase in TACI expression in HC total B cells, reaching the levels of dcSSc B cells. These changes can also be explained by the differences observed in naive B cells, because CD180 ligation augmented the expression of BAFF-R and reduced the expression of TACI in HC naive B cells, resulting in similar levels observed in the naive B cells of the dcSSc patients. 

Although our study carries the limitation that the number of patients enrolled was relatively low, it is an advantage that they formed a homogeneous group of early dcSSc cases. Therefore, we can conclude that B-cell activation via CD180 utilizes the PI3K/Akt/mTOR pathway in SSc and may contribute to a pathological shift in BAFF signaling, particularly in naive B cells, which adds to our previous findings proposing that the anti-CD180 antibody therapy suggested in SLE may not be beneficial in SSc. 

## 4. Materials and Methods

### 4.1. Patients

Table 2013. ACR/EULAR SSc classification criteria. Mean (SD) disease duration was 1.73 (±0.9) years based on the date of the first non-Raynaud’s symptom, mean (SD) age at enrollment was 49.88 (±15.6) years, mean (SD) modified Rodnan skin score was 16.42 (±11.9) points, and frequent internal organ involvements were interstitial lung disease (73.1%), gastrointestinal involvement (69.2%) and cardiac involvement (53.9%). The aforementioned internal organ changes corresponded to the involvement seen in the early phase of disease. The detailed characteristics of the patients are shown in Table 1. Controls (*n* = 33) were age- and sex-matched healthy individuals (HCs). All participants gave written informed consent to participate in the study, after approval by the Hungarian National Ethics Committee (ETT TUKEB 47861-6/2018/EKU).

### 4.2. Peripheral Blood Mononuclear Cell Isolation and B Cell Separation 

Peripheral blood mononuclear cells (PBMCs) were isolated by Ficoll-Paque Plus (GE Healthcare, Chicago, IL, USA) density gradient centrifugation of peripheral blood samples. The negative selection of B cells was performed using the MACS B cell isolation kit II (Miltenyi Biotech, Bergisch Gladbach, Germany), according to the manufacturer’s instructions. The purity of B cells was over 95%. 

### 4.3. Anti-CD180 Antibody Stimulation

For Phosflow measurements in dcSSc patients (*n* = 6) and HC (*n* = 7), 2 × 10^5^ B cells per condition were placed onto a 96-well plate in RPMI culture medium without FBS for 1 h. Then, the B cells were stimulated with LEAF purified anti-human CD180 (RP105) antibody (Clone: MHR73-11) (Bio-Legend, San Diego, CA, USA) at 1 µg/mL (anti-CD180) or left unstimulated for 30 min at 37 °C. To investigate the expression of BAFF-R and TACI of total B cells and B cell subsets in dcSSc (*n* = 4) and HCs (*n* = 4), 5 × 10^5^ PBMCs were stimulated with anti-CD180 antibody or left unstimulated for 24 h 37 °C.

### 4.4. RNA Isolation, cDNA Synthesis and qPCR for the Evaluation of BAFF-R Expression

To determine the BAFF-R mRNA expression of total B cells in dcSSc (*n* = 3) and HCs (*n* = 3), total RNA was extracted from isolated B cells using the NucleoSpin RNA XS kit (Macherey-Nagel Inc., Bethlehem, PA, USA). Following cDNA generation (High-Capacity cDNA Reverse Transcription Kit, Thermo Fisher Scientific, Waltham, MA, USA), the SensiFAST SYBR Lo-ROX Kit (Bioline, London, UK) was used to determine the BAFF-R mRNA expression of total B cells in dcSSc (*n* = 3) and HCs (*n* = 3). Amplifications were performed on the Applied Biosystems 7500 RT-PCR System (Thermo Fisher Scientific, Waltham, MA, USA). Gene expression was analyzed with 7500 Software v2.0.6 (Thermo Fisher Scientific, Waltham, MA, USA) and normalized to GAPDH (a “housekeeping” gene) as a reference. Fold changes (RQ) were calculated according to the 2-ddCT method.

### 4.5. Flow Cytometric Analysis of BAFF-R and TACI Expression

To measure the expression of BAFF-R and TACI of total B cells and B cell subgroups in dcSSc (*n* = 4) and HCs (*n* = 4), PBMCs were labeled using the combination of the following monoclonal antibodies: anti-human CD19-PE (SJ25C1, Becton Dickinson, Franklin Lakes, NJ, USA), anti-human IgD-PerCP (IA6-2, BioLegend, San Diego, CA, USA), anti-human CD27-APC (M-T271, Becton Dickinson, Franklin Lakes, NJ, USA), anti-human CD27-FITC (L128, Becton Dickinson, Franklin Lakes, NJ, USA) anti-human BAFF-R (CD268)-FITC (11C1, BioLegend, San Diego, CA, USA) and anti-human TACI (CD267)-APC (1A1, BioLegend, San Diego, CA, USA), following the manufacturer’s protocols. Briefly, PBMCs were incubated with the appropriate antibodies for 30 min on ice, washed twice in phosphate-buffered saline (PBS) and fixed with FACSFix (0.5% PFA in PBS). Fluorescence of the labeled cells was measured using a FACS Calibur flow cytometer (Becton Dickinson, Franklin Lakes, NJ, USA) and analyzed using FCS Express 6 software (De Novo Software, Pasadena, CA, USA).

### 4.6. Assessment of the Phosphorylation of Akt and S6

To analyze the phosphorylation of Akt (pS473) and S6 (pS235/pS236) of B cells in dcSSc (*n* = 6) and HCs (*n* = 7), we used the anti-human Akt (pS473)–Alexa Fluor488 (M89-61, Becton Dickinson, Franklin Lakes, NJ, USA) and S6 (pS235/pS236)–Alexa Fluor647 (N7-548, Becton Dickinson, Franklin Lakes, NJ, USA) antibody. Phosflow assay was performed in purified B cells according to the BD Phosflow Protocol, using BD Cytofix Fixation Buffer and BD Perm III Buffer (BD Biosciences, Franklin Lakes, NJ, USA). Briefly, after stimulation, cells were immediately fixed with pre-warmed Cytofix Fixation buffer for 10 min at 37 °C. Following washing, cells were permeabilized using pre-cooled Perm Buffer III for 30 min on ice. Then, the cells were washed three times and they were stained and were incubated for 30 min at room temperature. Afterwards, the cells were washed and immediately measured without fixation using a FACS Calibur flow cytometer (Becton Dickinson, Franklin Lakes, NJ, USA) and analyzed using FCS Express 6 software (De Novo Software, Pasadena, CA, USA).

### 4.7. Anti-BAFF Autoantibody Measurement 

The levels (mean fluorescence intensity, MFI) of autoantibodies against BAFF in the serum samples of dcSSc (*n* = 20) patients and HCs (*n* = 21) were determined using the MILLIPLEX Map Human Cytokine Autoantibody Panel (HCYTAAB-17K, Merck KGaA, Darmstadt, Germany) according to the manufacturer’s recommendations. The assay was run with the Luminex MAGPIX instrument (Luminex Corporation, Austin, TX, USA). Data were analyzed with Belysa Immunoassay Curve Fitting Software (Merck KGaA, Darmstadt, Germany).

### 4.8. Statistical Analysis

SPSS v. 27.0 statistics package (IBM, Armonk, NY, USA) was used for statistical evaluation using Student t-tests and Mann–Whitney U-test, where *p* values < 0.05 were considered significant.

## Figures and Tables

**Table 1:** Patients’ characteristics.

Characteristics	dcSSc Patients (*n* = 26)
Age (years), mean (SD)	49.88 (15.6)
Gender (female), *n* (%)	22 (84.6%)
Disease duration ^1^ (years), mean (SD)	1.73 (0.9)
Organ involvement	
MRSS mean (SD)	16.42 (11.9)
Lung fibrosis ^2^, *n* (%)	19/26 (73.1%)
Pulmonary arterial hypertension ^3^, *n* (%)	0/26 (0%)
Renal involvement ^4^, *n* (%)	1/26 (3.9%)
Gastrointestinal involvement ^5^, *n* (%)	18/26 (69.2%)
Cardiac involvement ^6^, *n* (%)	14/26 (53.9%)
Antibodies	
Anti-Scl-70+, *n* (%)	12/26 (46.2%)
Anti-RNA-polymerase III+, *n* (%)	4/26 (15.4%)
Immunosuppressive therapy ^7^, *n* (%)	17/26 (65.4%)

^1^ Onset of the disease was defined as the date of the first non-Raynaud’s symptom; ^2^ pulmonary fibrosis was characterized by detection of fibrosis with high-resolution CT and/or decreased forced vital capacity (FVC < 80%); ^3^ no signs suggestive of PAH on echocardiography and spirometry with DLCO determination; therefore, no right heart catheterization performed in patients; ^4^ scleroderma renal crisis was recorded as kidney involvement; ^5^ gastroesophageal involvement was established with barium swallow or esophago-gastroscopy; ^6^ cardiac involvement was defined by diastolic dysfunction or decreased left ventricular ejection fraction; ^7^ mycophenolate mofetil (*n* = 2), methotrexate (*n* = 4) or cyclophosphamide (*n* = 11).

## Data Availability

Data are contained within the article.
